# Supplementary material for: A Rapid Crosslinkable Maleimide-Modified Hyaluronic Acid and Gelatin Hydrogel Delivery System for Regenerative Applications
Source: Gels. 2021 Feb 1;7(1):13. doi: 10.3390/gels7010013 (PMC7931058; doi:10.3390/gels7010013)
Supplement: Supplementary file 1 [file gels-07-00013-s001.pdf]

Supplemental Materials for:

A Rapid Crosslinkable Maleimide-modified Hyaluronic Acid and Gelatin Hydrogel Delivery System for Regenerative Applications

Kyung Min Yoo[1], Sean Murphy[1,*], Aleksander Skardal[2,3,*],

[1]Wake Forest Institute for Regenerative Medicine, Wake Forest School of Medicine, 391 Technology Way, Winston-Salem, NC, 27101, USA.
[2]Department of Biomedical Engineering, The Ohio State University, Fontana Labs, 140 W. 19th Ave, Columbus, OH, 43210, USA.
[3]The Ohio State University and Arthur G. James Comprehensive Cancer Center, Columbus, Ohio, 43210, USA

*Correspondence:

Aleksander Skardal, PhD
Department of Biomedical Engineering,
The Ohio State University,
3022 Fontana Labs,
140 W. 19th Ave,
Columbus, OH, 43210, USA.

Sean V. Murphy, PhD
Wake Forest Institute for Regenerative Medicine,
Wake Forest School of Medicine,
391 Technology Way,
Winston-Salem, NC, 27101, USA.

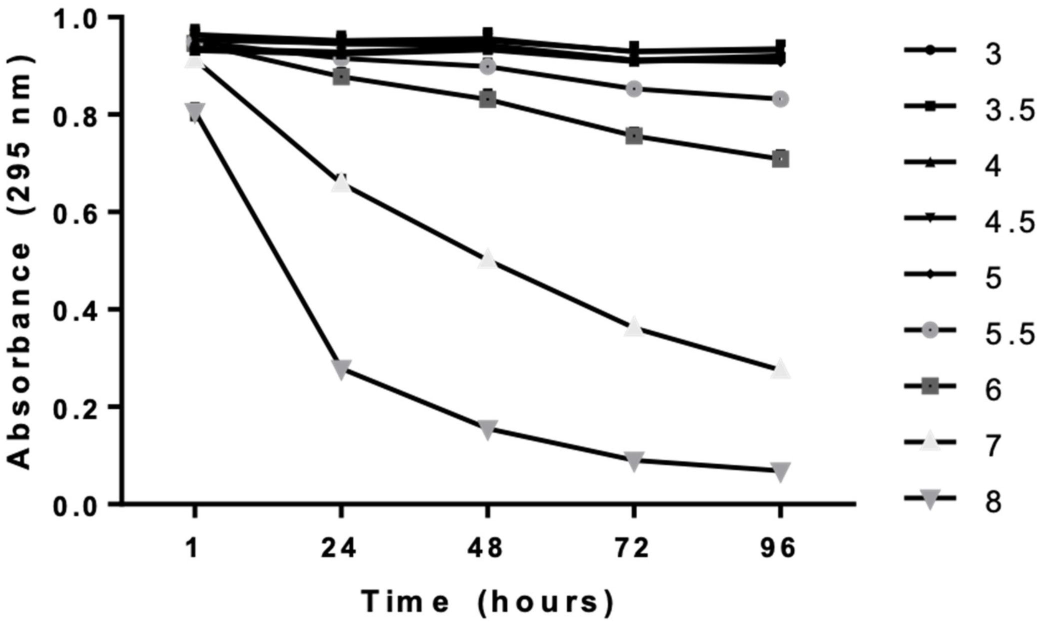

Supplementary Figure 1. Sensitivity of maleimides at various pH conditions (pH 3-8) using UV-vis Spectroscopy. Absorbances (n=3) per condition were measured at 295 nm.

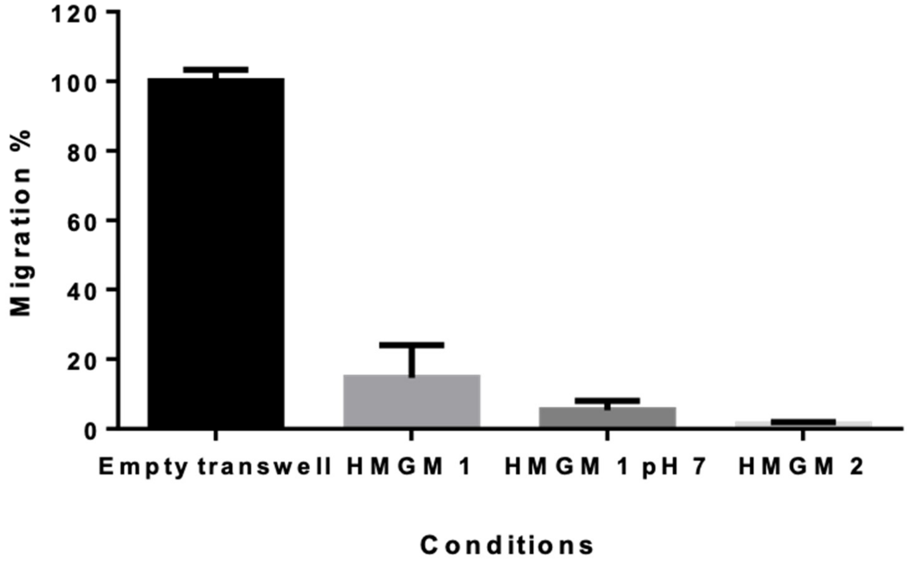

Supplementary Figure 2. Mechanical Barrier Assay with HMGM hydrogels. Migration percentages of FITC-dextran solution passed with hydrogels were calculated (n=3).

Hydrogel	Components	Final Conc. (w/v %)
HMGM 1	HA-Mal (100 kDa)	1.0%
	Gel-Mal (175 g)	0.4%
	PEGDSH (3.4 kDa)	0.25%
HMGM 2	HA-Mal (200 kDa)	0.5%
	Gel-Mal (300 g)	0.4%
	PEGDSH (3.4 kDa)	0.125%

Supplementary Table 1. Hydrogel composition.

HA-Mal MW (kDa)	HA-Mal Conc. (% w/v)	Gel-Mal bloom strength (g)	Gel-Mal Conc. (% w/v)	PEG Geometry	PEG MW (kDa)	PEG Conc (% w/v)	Stiffness Score (1-5)	Easily Extrudable	Self Standing
100	1	175	1.6	2 arm	3.4	0.5	3.5	x	x
100	1	175	1.6	2 arm	3.4	1	3.25	x	x
100	1	175	1.6	4 arm	10	1	3.75		x
100	2	175	1.6	2 arm	3.4	0.5	4	x	x
100	2	300	1.6	2 arm	3.4	0.5	4	x	x
200	1	175	1.6	2 arm	3.4	0.5	4	x	x
200	1	175	1.6	4 arm	10	1	3.25		x
200	1	175	1.6	2 arm	3.4	0.5	4	x	x
200	1	300	1.6	2 arm	3.4	0.5	4	x	x
200	1	300	1.6	2 arm	3.4	0.5	4	x	x

Supplementary Table 2. Hydrogel formulations tested for ability to self-stand and for ease of extrusion. “x” indicates a positive result in terms of extrusion or ability for self-support.

Swelling %

	t=0	t=1	t=2	t=3	t=6	t=24
HMGM 1	0	10 ± 3%	14 ± 4%	22 ± 13%	25 ± 12%	32 ± 13%
HMGM 2	0	23 ± 19%	27 ± 13%	21 ± 10%	24 ± 11%	26 ± 9%

Supplementary Table 3. Swelling table.
